# A Pilot Study of the Effects of Mindfulness-Based Cognitive Therapy on Positive Affect and Social Anxiety Symptoms

**DOI:** 10.3389/fpsyg.2018.00866

**Published:** 2018-06-01

**Authors:** Marlene V. Strege, Deanna Swain, Lauren Bochicchio, Andrew Valdespino, John A. Richey

**Affiliations:** Department of Psychology, Virginia Tech, Blacksburg, VA, United States

**Keywords:** social anxiety disorder, MBCT, positive affect, negative affect, mindfulness

## Abstract

Randomized controlled trials have demonstrated that mindfulness-based cognitive therapy (MBCT) is efficacious in reducing residual depressive symptoms and preventing future depressive episodes (Kuyken et al., 2016). One potential treatment effect of MBCT may be improvement of positive affect (PA), due to improved awareness of daily positive events (Geschwind et al., 2011). Considering social anxiety disorder (SAD) is characterized by diminished PA (Brown et al., 1998; Kashdan, 2007), we sought to determine whether MBCT would reduce social anxiety symptoms, and whether this reduction would be associated with improvement of PA deficits. Adults (*N* = 22) who met criteria for varied anxiety disorders participated in a small, open-label trial of an 8-week manualized MBCT intervention. Most participants presented with either a diagnosis (primary, secondary, or tertiary) of generalized anxiety disorder (GAD) (*N* = 15) and/or SAD (*N* = 14) prior to treatment, with eight individuals meeting diagnostic criteria for both GAD and SAD. We hypothesized participants would demonstrate improvements in social anxiety symptoms, which would be predicted by improvements in PA, not reductions in negative affect (NA). Results of several hierarchical linear regression analyses (completed in both full and disorder-specific samples) indicated that improvements in PA but not reductions in NA predicted social anxiety improvement. This effect was not observed for symptoms of worry, which were instead predicted by decreased NA for individuals diagnosed with GAD and both decreased NA and increased PA in the entire sample. Results suggest that MBCT may be efficacious in mitigating social anxiety symptoms, and this therapeutic effect may be linked to improvements in PA. However, further work is necessary considering the small, heterogeneous sample, uncontrolled study design, and exploratory nature of the study.

## Introduction

Social anxiety disorder (SAD) is a common and debilitating psychiatric disorder marked by fear of one or more social situations (American Psychiatric Association, 2013). Current treatment approaches for SAD focus mainly on fear extinction and habituation processes as their primary mechanisms of action (Rodebaugh et al., 2004). However, poor treatment response rates have been observed for SAD, with a considerable proportion of individuals still reporting clinically significant symptoms after completing treatment (Heimberg et al., 1998; Otto et al., 2000). Thus, research exploring alternative modes of intervention for SAD is needed. Recent evidence suggests that unlike other anxiety disorders, SAD may be uniquely characterized by anhedonic symptoms and low positive affect (PA; Brown et al., 1998; Hughes et al., 2006; Kashdan, 2007). Mindfulness-based cognitive therapy (MBCT) appears to mitigate PA deficits (Schroevers and Brandsma, 2010; Geschwind et al., 2011), and has been gaining empirical support for use in anxiety and mood disorder populations (Hofmann et al., 2010; Fjorback et al., 2011). Accordingly, the current study utilized an 8-week manualized MBCT protocol in a sample of adults with anxiety disorders, to examine the role of PA change in social anxiety symptom reduction relative to reduction in other anxiety symptoms.

Relatively early evidence for SAD-related deficits in PA was reported by Brown et al. (1998), who examined quantitative structural models incorporating latent factors pertaining to NA, PA, and autonomic arousal in relation to symptoms of several anxiety disorders (SAD, GAD, panic disorder, obsessive-compulsive disorder) and depression. In the best fitting model, SAD appeared to be unique among the anxiety disorders in that it is associated with lower PA in addition to higher NA. This ran contrary to the influential and comparatively earlier *tripartite model* account, which posited that all anxiety disorders are associated with increased NA and autonomic arousal, but not deficits in PA (Clark and Watson, 1991). More recently, Kashdan (2007) expanded upon this by conducting a meta-analysis on the relationship between PA with social anxiety symptoms, finding an estimated correlation of -0.36 across studies. Further support comes from neuroimaging research, which suggests that SAD is associated with diminished activation of brain regions associated with reward during the anticipation of social, but not monetary incentives (Richey et al., 2014, 2017). Thus, there is increasing recognition, both in the behavioral and neurological domains that social anxiety is associated not only with anxious arousal, but also with dysfunctional approach-related motivation and decreases in PA. To the extent that current treatments do not focus on PA deficits in SAD, it is therefore possible that separate and identifiable mechanisms of pathophysiology may serve to maintain the disorder, but are not incorporated into current mainstream treatment approaches. Therefore, additional research is needed to clarify whether modifying this target could be beneficial in reducing symptoms of SAD. Given its demonstrated effectiveness in improving PA (Geschwind et al., 2011), MBCT may be an efficacious alternative treatment approach for reducing SAD symptoms.

As MBCT was originally developed as a therapeutic intervention to reduce the likelihood of depression relapse, the intervention’s underlying theory pertains to the cognitive mechanisms believed to result in repeated depressive episodes. Teasdale (1988) posited that during repeated depressive episodes, dysphoric mood states and rumination become fused in a cycle of reciprocal cause and effect, such that a drop in mood automatically activates the associated maladaptive thinking pattern, which can then result in another depressive episode. Thus, negative mood and rumination maintain a joint linkage to major depressive disorder by virtue of their mutual association. MBCT is believed to mitigate the association between dysphoric mood and a ruminative response style by replacing rumination instead with mindful attention (Lau et al., 2004; Segal et al., 2012). This mindful attention is thought to prevent the reinstatement of maladaptive thinking patterns specifically by dissociating cognitions from their emotional content. Thus, individuals who practice mindful awareness may be able to break the thought-affect cycle in which deteriorations of mood results in rumination and vice versa. Recent work (Batink et al., 2013; Gu et al., 2015; van Aalderen et al., 2015) appears to support this conceptualization of mindfulness-based intervention mechanisms, in which emotional reactivity is specifically mitigated by MBCT, leading the individual to detect small deteriorations in mood and address this drop in mood with ameliorative action. In turn, this is thought to prevent decline into another full depressive episode (Segal et al., 2012).

In addition to an increased regulatory capacity for NA, evidence suggests that MBCT may also improve positive emotional tone. For example, Geschwind et al. (2011) proposed that MBCT may improve PA in parallel with decreases in NA, as the mindful awareness associated with the therapeutic intervention may also facilitate identification of daily, albeit fleeting positive emotions, which prior to intervention may go unnoticed. Moreover, MBCT may improve the ability to experience or savor positive emotions specifically by promoting a curiosity and openness toward emotional experience. To test the hypothesis that MBCT has a distinct impact on positive emotional experience, Geschwind and colleagues randomly assigned 130 individuals with residual depression symptoms to a MBCT treatment condition or a waitlist control condition. Throughout the study, participants were asked to regularly report on their current daily experiences (the pleasantness of an activity and their affect), at randomly selected times each day. These ratings also yielded a specific estimate of the rewarding effect of the experience, calculated as the effect of the pleasantness of the experience on state PA. Results indicated that MBCT was associated with increased values on all three ecological momentary measures (pleasantness, affect, and rewarding effect of experience) as compared to waitlist. Moreover, improvements in these measures of PA appear to also have a specific impact on residual depressive symptoms.

Given that MBCT appears to bolster PA, and social anxiety is associated with PA deficits, it therefore follows that MBCT may also be an efficacious intervention in this population. In support of this reasoning, a recent randomized controlled trial of 26 adults with SAD (Piet et al., 2010) compared cognitive behavioral therapy (CBT) in a group therapy format (GCBT; *N* = 12; Heimberg and Becker, 2002) to MBCT, and found that MBCT (*N* = 14) was effective in improving SAD symptoms, although less so than GCBT (Cohen’s *d* = 0.78 and 1.15 respectively, on a composite measure of SAD symptoms at post-treatment). The authors concluded that the effect size for the MBCT group while numerically smaller than that observed for the GCBT group was not statistically different, thus illustrating that MBCT may have promise as an alternative method of addressing SAD symptoms.

While promising, results from Piet et al. (2010) and related studies do not assess mechanistic predictors of change when MBCT is applied to social anxiety symptoms, and more specifically do not assess the relationship between PA and social anxiety improvement. Accordingly, the purpose of the current study was to determine whether MBCT might be efficacious in mitigating SAD symptoms and whether this symptom improvement would be linked to improvements in PA. Given the lack of research pertaining to this topic, our study was exploratory in nature. From this limited literature base, we provide the following preliminary hypotheses: (1) participants treated with MBCT would demonstrate social anxiety symptom improvement at post-treatment, as measured by reductions in self-reported social anxiety symptoms, (2) improvement in social anxiety symptoms, but not other anxiety symptoms would be uniquely related to PA change, and (3) improvements in mindful awareness would be related to improvements in PA change, as MBCT is believed to improve one’s ability to detect daily positive experiences (Geschwind et al., 2011). To test these hypotheses, we conducted a small, uncontrolled pilot trial of MBCT. Twenty-two adults completed treatment, 14 of which had a diagnosis of SAD. This heterogeneous sample allowed for the comparison between anxiety disorders on the relationship between PA improvements and symptom reduction.

## Materials and Methods

### Recruitment and Screening

All participants provided written informed consent, in accordance with the provisions of the Declaration of Helsinki, and as approved by the Virginia Tech institutional review board (IRB). Participants were recruited via online advertisements as well as flyers posted throughout the community. Advertisements introduced the study as a potential treatment for improving one’s anxiety, with a special emphasis on social anxiety. Interested individuals completed a brief phone screening during which their anxiety symptoms and medication history were assessed for potential eligibility, and participants were informed of the nature of the treatment and study. Participants then underwent a diagnostic interview administered by a doctoral student clinician. Participants were deemed eligible if they met diagnostic criteria for an anxiety disorder as determined by the Anxiety Disorder Interview Schedule for DSM-IV (ADIS-IV, Brown et al., 1994) and were not currently undergoing therapy for anxiety. Participants taking psychotropic medication were not excluded, provided the dosage had remained constant for 1 month prior to the start of the group.

### Participants

**FIGURE 1:**
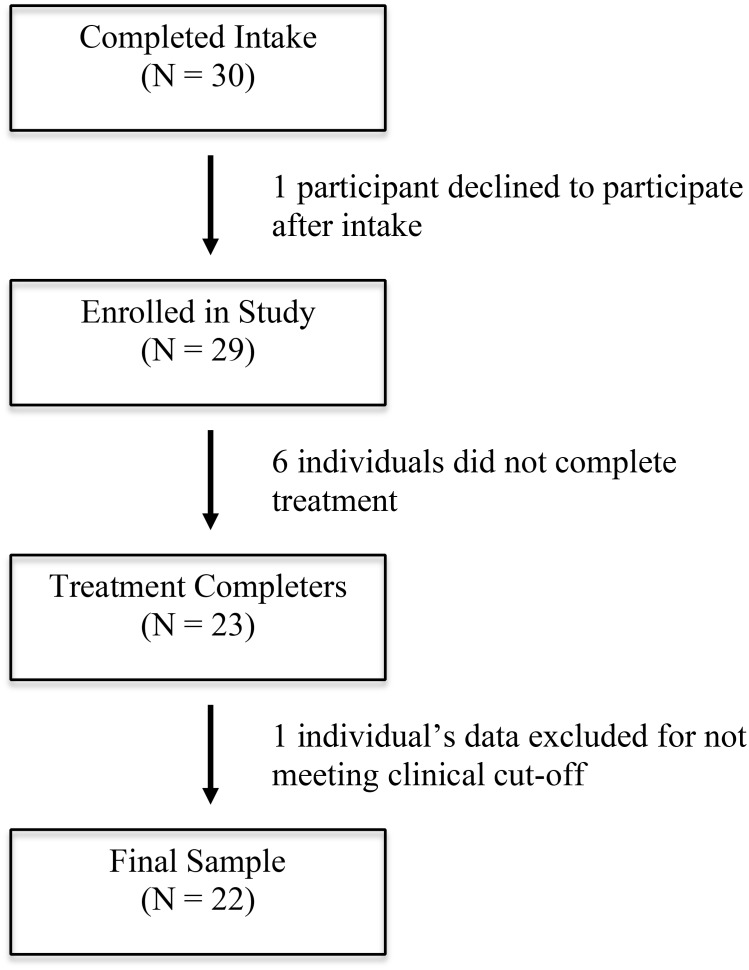
Consort diagram of study participants.

Of the initial 30 individuals who completed an intake interview, 29 were enrolled in the study, and these individuals took part in one of three separate groups conducted sequentially over 18 months (Group 1: *N* = 9; Group 2: *N* = 8; Group 3: *N* = 12). One participant who participated in the third group only met subclinical levels of generalized anxiety. This participant was allowed to participate, but their data was not included in the final analyses. An additional six participants (two from each of the three groups) dropped out of the study prior to group completion and thus did not complete the post-treatment assessment. This resulted in a final sample of 22 participants (see **Figure 1** for consort diagram). Group members were primarily female and Caucasian (see **Table 1** for demographics of each group), and the most common principal diagnoses were SAD and GAD (see **Table 2** for diagnoses at pre-treatment).

**Table 1:** Demographics of treatment groups.

	Groups		
	1	2	3
Gender	∼57% female	50% female	70% female
Age	28.29 (12.27)	35.00 (13.27)	42.50 (12.50)
Ethnicity	∼70% Caucasian	100% Caucasian	100% Caucasian

*Group 1 (N = 7), Group 2 (N = 6), Group 3 (N = 10). Age is represented as mean years followed by standard deviation.*

**Table 2:** Primary, secondary, and tertiary diagnoses.

	Primary	Secondary	Tertiary
Social Anxiety	8	4	2
Generalized Anxiety	10	5	0
Panic Disorder/Agoraphobia	2	1	2
Obsessive–Compulsive Disorder	0	1	1
Post-Traumatic Stress Disorder	1	0	0
Specific Phobia	0	3	1
Persistent Depressive Disorder	1	1	2

*Numbers represent total number of individuals in the completer sample (N = 22) with that diagnosis prior to treatment.*

### Diagnostic Interview

The Anxiety Disorder Interview Schedule for DSM-IV (Brown et al., 1994) is a semi-structured diagnostic interview that assesses an individual for symptoms pertaining to a selection of disorders present in the DSM-IV, namely anxiety disorders, depression, and substance abuse and dependence. The ADIS-IV was conducted for each participant prior to the group intervention and once again after the intervention. Doctoral student clinicians, who underwent reliability training on the ADIS-IV prior to administration, conducted the interviews. ADIS-IV reliability training required that assessors first observe live interviews of the administration of the ADIS-IV by a research-reliable assessor. Subsequently the trainee conducted the clinical interview while being observed by a research-reliable assessor. These interviews were recorded and scored live, with diagnoses as well as clinical severity ratings (CSR) being compared between the trainee and reliable assessor subsequent to each interview. To achieve research reliability, assessors were required to match the trainer on principal as well as co-occurring diagnoses on three interviews (±1 CSR) before administering the ADIS-IV independently.

### Questionnaires

#### Liebowitz Social Anxiety Scale – Self-Report (LSAS-SR, Liebowitz, 1987)

The LSAS-SR is a 24-item self-report scale that measures characteristic symptoms of social anxiety. There are 11 items related to social interaction and 13 related to public performance. The participants are asked to rate their fear and avoidance of these situations on a 4-point scale, with 0 being no fear or never avoid and 3 being severe fear or usually avoid. The fear and avoidance scores are summed for a total score. The scale has demonstrated strong test–rest reliability in a clinical sample (Baker et al., 2002). In the current study, the measure’s internal consistency was acceptable; the Cronbach’s alphas were 0.96 for pre-treatment and 0.95 for post-treatment.

#### Positive Affect and Negative Affect Schedule (PANAS, Watson et al., 1988)

The PANAS is a brief, 20-item self-report measure to determine positive and negative affect (NA). Watson et al. (1988) describe PA as the extent to which an individual feels energetic, accomplished, and motivated, with higher scores indicating more positive feelings. A high NA score reflects an individual experiencing unpleasant or distressing feelings. Subjects rate to what extent each of the 20 items describes their current mood from 1 (very slightly or not at all) to 5 (extremely). Two separate scores are assigned for positive (PANAS-PA) and negative affect (PANAS-NA), ranging from 10 to 50. Cronbach’s α for both the PA and NA scales suggested excellent internal consistency, [PANAS-PA pre (0.92) and post (0.95), PANAS-NA pre (0.95) and post (0.93)].

#### Penn State Worry Questionnaire – Self-Report (PSWQ, Meyer et al., 1990)

The PSWQ is a 16-item self-report measure that assesses worry. Items are scored on a Likert-type scale from 1 (*not at all typical of me*) to 5 (*very typical of me*), with five questions reverse scored. The PSWQ has demonstrated strong reliability and validity, and has been shown to allow for the discrimination of GAD from other anxiety disorders (Hazlett-Stevens et al., 2004). The PSWQ demonstrated good internal consistency in the current sample (PSWQ pre α = 0.91, PSWQ post α = 0.90).

#### Five Facet Mindfulness Questionnaire – Self-Report (FFMQ, Baer et al., 2008)

The FFMQ is a 39-item self-report measure that yields overall and categorical scores for mindfulness. There are five subscales: observing (being aware of one’s surroundings), describing (being able to explain how one feels at a given time), acting with awareness (being in the present and taking notice of one’s actions), non-judging of inner experience (being aware of one’s emotions, without experiencing guilt for feeling a certain way), and non-reactivity to inner experience (being aware of one’s emotions without feeling a need to react to them). Items are scored from 1 (*never or very rarely true*) to 5 (*very often or always true*). The FFMQ has demonstrated good internal consistency, with Cronbach’s α ranging from 0.85 to 0.90 for non-meditating and meditating validation samples respectively (de Bruin et al., 2012). The FFMQ has also exhibited good construct validity among both meditators and non-meditators (Baer et al., 2008). FFMQ demonstrated good internal consistency in the current sample (FFMQ pre α = 0.82, FFMQ post α = 0.89). Individual subscales showed adequate internal consistency, with pre α’s ranging from 0.73 to 0.97 and post α’s ranging from 0.71 to 0.96.

### Procedure

After completing the initial diagnostic interview and questionnaires, participants were enrolled in one of the three MBCT therapy groups. Three separate groups took place over the course of 18 months and were led by a Ph.D.-level clinical psychologist trained in MBCT (J.A.R.). The protocol followed Segal et al. (2012) manual for MBCT for Depression. Under this protocol, participants were asked to attend 8 weekly, 2-h group meetings, during which the group leader led participants in mindfulness and meditation exercises. On average, participants attended 5.39 (*SD* = 2.46) sessions. The participants were assigned homework exercises to complete outside of treatment, involving similar mindfulness and meditation practices introduced during the weekly sessions. We advised participants to complete these exercises daily. Materials (handouts and audio tracks) were available on a website for the individuals to access throughout each week. Within 1 week of completion of the program, participants attended a post-treatment assessment, where they underwent another diagnostic interview (ADIS-IV; Brown et al., 1994) with a ADIS-IV reliable doctoral student clinician and filled out the post-treatment questionnaires.

### Statistical Analyses

To assess whether MBCT would result in improvements in self-reported social anxiety symptoms, we conducted two paired-samples *t*-tests (pre- *vs.* post-treatment), in the entire sample and in the more selective group consisting only of participants with a SAD diagnosis at the start of treatment (*N* = 14). In order to test our hypothesis that change in PA would uniquely predict change in social anxiety symptoms, we conducted a series of three-step hierarchical multiple regression analyses, first predicting social anxiety symptoms after treatment (LSAS-SR), then for comparison, predicting GAD worry (PSWQ) after treatment. For each model, we entered pre-treatment anxiety symptoms (LSAS-SR or PSWQ), negative affect (PANAS-NA), and positive affect (PANAS-PA) at the first level to account for baseline anxiety symptoms and affect. At the second level, we entered change in NA (subtracting post from pre PANAS-NA scores) to first account for anxiety symptom change associated with reduction in NA. At the final level, we entered PA change (subtracting post from pre PANAS-PA scores), to assess the influence of PA change on anxiety reduction. All independent variables were mean centered prior to their inclusion in the analyses. The separate social anxiety and GAD worry regressions were first conducted using the entire sample of participants who completed treatment (*N* = 22). Then, we examined the same model configuration in cases with a SAD pre-treatment diagnosis (*N* = 14), and finally for individuals with a pre-treatment diagnosis of GAD (*N* = 15). There were eight individuals who had co-occurring SAD and GAD prior to treatment, thus these individuals were included in both the SAD and GAD analyses.

In order to examine the relationship between mindful awareness and improvements in PA, we first conducted a regression with change in mindful awareness (awareness subscale of the FFMQ) predicting PA change in the full sample. To further assess whether the relationship between mindful awareness change and PA change is unique among other facets of mindfulness, we conducted a multiple regression, with change scores for each FFMQ subscale (observing, describing, acting with awareness, non-judging of inner experience, and non-reactivity to inner experience) predicting PA change.

## Results

### Hypothesis 1: MBCT Improves Social Anxiety Symptoms

In the entire sample, results indicated a significant reduction in LSAS-SR total score at post-treatment, with a medium effect size. In addition, participants with a diagnosis of SAD (*N* = 14), also reported a significant reduction in social anxiety symptoms with a medium effect size, see **Table 3** for these and other pre-post comparisons of questionnaire data. Thus, the pattern of pre- to post-treatment change in LSAS-SR symptoms supported hypothesis 1, to the extent that SAD symptoms were significantly reduced by the MBCT intervention. Of the 14 individuals with SAD at the start of treatment, six no longer met diagnostic criteria for SAD after treatment, see **Table 4** for all anxiety diagnoses before and after treatment.

**Table 3:** Changes in self-report variables.

		Pre-MBCT	Post-MBCT	*t*-value	*p*-value	Effect size
Measure/Sample	*N*	*M* (*SD*)	*M* (*SD*)	*t*	*p*	*d*
**LSAS-SR**						
Full-Sample	22	60.95 (29.25)	47.68 (23.63)	3.26	0.004	0.45
SAD	14	68.86 (25.54)	52.86 (22.22)	2.65	0.020	0.63
**PSWQ**						
Full-Sample	22	52.05 (16.06)	45.36 (14.54)	2.81	0.010	0.42
GAD	15	54.33 (14.34)	46.40 (15.44)	3.02	0.009	0.55
**PANAS: PA**						
Full-Sample	22	20.41 (8.59)	24.59 (10.46)	-3.06	0.006	0.49
SAD	14	20.64 (9.54)	25.50 (11.89)	-2.85	0.014	0.51
GAD	15	19.47 (7.97)	23.07 (7.78)	-2.57	0.022	0.45
**PANAS: NA**						
Full-Sample	22	13.27 (10.57)	10.23 (7.80)	2.12	0.046	0.29
SAD	14	15.57 (11.96)	11.29 (7.61)	2.08	0.058	0.36
GAD	15	14.47 (11.61)	10.60 (8.25)	2.05	0.060	0.33
**FFMQ: Awareness**						
Full-Sample	22	24.86 (6.79)	28.00 (6.36)	-2.90	0.009	0.46
SAD	14	24.43 (6.66)	28.43 (6.42)	-2.67	0.019	0.60
GAD	15	24.00 (7.45)	26.73 (6.87)	-2.05	0.059	0.37
**FFMQ: Total**						
Full-Sample	22	109.86 (17.46)	123.50 (21.50)	-3.64	0.002	0.78
SAD	14	109.07 (17.08)	129.93 (18.82)	-4.95	<0.001	1.22
GAD	15	108.67 (17.88)	120.87 (18.31)	-2.51	0.025	0.68

*SAD, Individuals with social anxiety disorder diagnosis; GAD, Individuals with generalized anxiety disorder diagnosis; LSAS-SR (Liebowitz Social Anxiety Scale – Self-Report; Liebowitz, 1987); PSWQ (Penn State Worry Questionnaire – Self-Report; Meyer et al., 1990); PANAS, PA/NA (Positive Affect/Negative Affect subscales of Positive Affect and Negative Affect Schedule; Watson et al., 1988); FFMQ, Awareness/Total (Awareness subscale and Total scale of the Five Facet Mindfulness Questionnaire – Self-Report; Baer et al., 2008); Effect size (d) = difference in means/pre-treatment standard deviation.*

**Table 4:** Anxiety diagnoses prior to and after treatment.

	Pre	Post
Social Anxiety	14	8
Generalized Anxiety	15	5
Panic Disorder/Agoraphobia	5	3
Obsessive–Compulsive Disorder	2	1
Post-Traumatic Stress Disorder	1	1
Specific Phobia	4	2

*Pre numbers represent total number of individuals who met criteria for an anxiety diagnosis prior to treatment. The post column represents the number of those individuals who still met diagnostic criteria after treatment.*

### Hypothesis 2: Social Anxiety Improvement Is Uniquely Predicted by Positive Affect Change

#### Predicting SAD Symptom Reduction: Full Sample

Results of the three-step hierarchical regression predicting SAD symptom reduction in the full sample revealed that the block one (LSAS-SR, *β* = 0.77, *p* = 0.001; PANAS-NA, *β* = 0.14, *p* = 0.631; PANAS-PA, *β* = -0.07, *p* = 0.677) indicated a significant increase in variance explained [*R*^2^ = 0.58, *FΔ*(3,18) = 8.13, *p* = 0.001]. Block two (NA change) did not result in a significant increase in variance explained [*R*^2^= 0.04, *FΔ*(1,17) = 1.55, *p* = 0.230]. The addition of block three (PA change, *β* = 0.34, *p* = 0.035) resulted in a significant change in *R*^2^ = 0.10, *FΔ*(1,16) = 5.30, *p* = 0.035, suggesting that PA change was predictive of LSAS-SR post treatment scores, beyond both baseline measures as well as NA change.

#### Predicting GAD Symptom Reduction: Full Sample

Results of the three-step hierarchical regression predicting GAD worry symptoms in the entire sample revealed that block one (PSWQ, *β* = 0.27, *p* = 0.241; PANAS-NA, *β* = 0.76, *p* = 0.026; PANAS-PA, *β* = -0.01, *p* = 0.961) resulted in a significant change in *R*^2^(0.55), *FΔ* (3,18) = 7.31, *p* = 0.002). Block two [PANAS: NA change, *β* = -0.62, *p* = 0.018) also resulted in a significant change in *R*^2^, *FΔ*(1,17) = 8.05, *p* = 0.011], explaining an additional 14.5% of the variance in post-treatment PSWQ. The addition of block three (PANAS: PA change, *β* = 0.30, *p* = 0.026) likewise significantly improved *R*^2^, *FΔ*(1,16) = 6.04, *p* = 0.026, explaining an additional 8.4% of the variance in post-treatment worries. Thus, results of this analysis indicated that both NA and PA change were significantly related to GAD-related symptoms, as measured by the PSWQ.

#### Predicting SAD Symptom Reduction: SAD-Specific Sample

Results of hierarchical regression predicting SAD symptoms in the SAD-specific sample revealed that neither the inclusion of the first (pre LSAS-SR, PA, and NA) or second (NA change) block resulted in a significant change in *R*^2^, as before, the inclusion of the third block (PA change, *β* = 0.58, *p* = 0.018) predicted a significant additional 30% of the variance in post-treatment social anxiety symptoms, *FΔ*(1,8) = 8.72, *p* = 0.018.

#### Predicting GAD Symptom Reduction: GAD-Specific Sample

Results of the three-step hierarchical regression predicting GAD worry symptoms in the GAD-specific sample show that the inclusion of the first block (PSWQ, *β* = 0.12, *p* = 0.705; PANAS-NA, *β* = 0.86, *p* = 0.088; PANAS-PA, *β* = 0.26, *p* = 0.208) resulted in a *R*^2^ change of 0.66, which was statistically significant, *FΔ*(3,11) = 7.09, *p* = 0.006. The inclusion of the second block (NA change, *β* = -0.84, *p* = 0.038) predicted an additional 14.7% of the variance in post-treatment worry symptoms, *FΔ*(1,10) = 7.55, *p* = 0.021. However, the third block (PA change) did not result in a significant *R*^2^ change (<0.01), *FΔ*(1,9) = 0.14, *p* = 0.714.

### Hypothesis 3: Improvements in Mindful Awareness Are Related to Improvements in PA

Consistent with this hypothesis, improvements in mindful awareness significantly predicted 26% of the variance in PA change [*β* = 0.51, *F*(1,21) = 6.84, *p* = 0.017]. However, when other aspects of mindfulness were included as predictors in the regression, the overall model was not significant, [*R*^2^= 0.36, *F*(5,16) = 1.83, *p* = 0.163], with no unique predictors (all *p’s* > 0.2). We also assessed whether change in the total FFMQ mindfulness score (the summation of all the subscales) predicted PA change in a simple regression. Change in FFMQ total score did not significantly predict PA change [*R*^2^= 0.18, *F*(1,20) = 4.32, *p* = 0.051]. Thus, there is only partial support of the hypothesis that improvements in mindful awareness are related to improvements in PA.

## Discussion

The purpose of the current study was to evaluate the relationship between PA and social anxiety symptom change in the context of a pilot open-label trial of MBCT. Consistent with initial predictions, results suggested that MBCT reduces SAD symptoms, and changes in positive, but not NA, appear to be linked to symptom reduction. Specifically, change in social anxiety symptoms from pre- to post-treatment was predicted by PA change both in the entire sample, and when constrained to only cases with a diagnosis of SAD. We also found that symptoms of worry in the GAD-specific sample were predicted exclusively by NA change, not NA and PA change, as was the case for symptoms of worry in the total sample. Thus, a more fine-grained analysis including only those with the symptom-related diagnosis (SAD and GAD, respectively) indicated that PA change appeared to be more closely related to SAD symptom improvement, and was less related to improvement in worry, when evaluated solely in the context of cases with a diagnosis of GAD. Moreover, this improvement in PA appears to be associated with improvements in mindful awareness. Overall, given that many individuals with SAD who undergo traditional, exposure-based interventions still experience clinically-significant symptoms after treatment (Heimberg et al., 1998; Otto et al., 2000), results of this study are promising in that they suggest that SAD symptoms may be addressed through alternative means, by increasing mindful awareness and improving PA.

Most prior work in MBCT has focused on depressed or remitted depressed patients (Teasdale et al., 2000; Ma and Teasdale, 2004; Kuyken et al., 2008), thus the application of MBCT to social anxiety remains a new area of inquiry. Piet et al. (2010) published the only clinical trial of MBCT in a socially anxious sample, and found that MBCT was effective in improving social anxiety symptoms. Results from the current study support their finding, as participants experienced a reduction in social anxiety symptoms after undergoing MBCT. Our study further expands upon the results reported by Piet et al. (2010), who did not assess for potential MBCT mechanisms of symptom improvement. Instead, they reasoned that MBCT would improve SAD symptoms by improving mindfulness, which would then aid in the regulation of NA. While null NA results should be interpreted with caution in the context of the current pilot study, our results do suggest that changes in PA may be relatively more associated with social anxiety symptom improvements.

Consistent with Brown et al. (1998), who found that social anxiety is related to reduced PA, we found that improvements in PA were predictive of improvements in social anxiety symptoms. The current study expands upon earlier work by suggesting that MBCT may be an effective treatment modality for addressing PA deficits and improving social anxiety symptoms. Although Brown and colleagues also found enhanced NA in SAD, we did not find that improvements in NA were predictive of improvements in social anxiety symptoms. Thus, our results indicated that MBCT may be relatively more suited for targeting PA in individuals with SAD. In addition, we found that a reduction in NA was predictive of improvements in worry, both in the full sample and GAD-specific sample. This is consistent with the broader literature linking NA to worry (e.g., Brown et al., 1998; McLaughlin et al., 2007). Although PA deficits are not believed to be related to GAD (Brown et al., 1998), we found that improvements in PA were predictive of improvements in worry in the full sample. This finding might be related to characteristics of our primary measure of worrying, the PSWQ. This measure may be sensitive to certain social anxiety symptoms with questions such as, “Many situations make me worry.” This possibility is consistent with our finding that the relationship between increased PA and worry improvement disappears when examined in the GAD-specific sample. The absence of the relationship between worry and PA in the GAD-specific sample could also be due to the smaller sample size; however, the NA relationship with worry was statistically significant in the GAD-specific sample.

There was partial support for the hypothesized link between improvements in mindful awareness and increased PA. Specifically, a simple regression indicated an expected association between change in mindful awareness and PA change. However, this association failed to reach significance when considered in the context of additional mindfulness subscale covariates. An association between mindful awareness and PA change is consistent with Geschwind et al. (2011) who proposed that MBCT increases PA by cultivating awareness, which then allows one to better notice daily positive experiences one might encounter that traditionally may be more likely to go unnoticed. It is also consistent with the literature suggesting that social anxiety symptoms are associated with reduced awareness in-the-moment, in favor for greater post-event ruminative processing (see Kashdan et al., 2011, for review). This motivated our initial hypothesis regarding change in mindful awareness as it might relate to specific improvements in PA. It should also be noted though, that when examining all facets of mindfulness as measured by FFMQ subscales (observing, describing, acting with awareness, non-judging of inner experience, and non-reactivity to inner experience), no subscale besides mindful awareness stood out as a unique predictor. This null result may be due to a number of reasons, including conceptual overlap among the mindfulness facets measured as well as lack of power due to the small sample size. Change in the FFMQ total score was not a significant predictor of PA improvement, which also may be in part due to the reduced power associated with the small sample size.

To our knowledge, the current study, along with the investigation by Piet et al. (2010) are the only studies that examine the impact of MBCT on social anxiety. However, several prior studies have examined the relationship between social anxiety symptoms and mindfulness-based approaches (e.g., Bögels and Mansell, 2004 for review; Kocovski et al., 2009), with a particular wealth of studies examining mindfulness-based stress reduction (MBSR; Ludwig and Kabat-Zinn, 2008; Khoury et al., 2015). MBSR was originally developed for chronic pain, stress and illness, and in a number of studies has also been evaluated as a direct intervention for social anxiety symptoms (Koszycki et al., 2007; Irving et al., 2009; Goldin and Gross, 2010; Piet et al., 2010). As noted in a recent review and meta-analysis by Gu et al. (2015), a consensus has not yet been reached regarding the similarities and differences between MBSR and MBCT. However, they appear to be similar in their general core principles, including (1) a focus on mindfulness practice both in-session and at home, (2) the highly systematized delivery of treatment modules through manualization, and (3) the specific application of mindfulness techniques to “be with” or experience difficult emotions. Goldin and Gross (2010) reported in a neuroimaging study of MBSR that a likely mechanism of action pertains to reductions in aversive arousal during feared situations, perhaps including reduced amygdala activity in response to negative self-beliefs (Goldin and Gross, 2010). In a more recent clinical trial, Goldin et al. (2016) compared MBSR to GCBT (Heimberg and Becker, 2002), and examined the cognitive and behavioral mediators of change in these two approaches. Results suggested that GCBT and MBSR may share multiple psychological mechanisms of change, including improvements in avoidance behaviors, reappraisal frequency, attention focusing and attention shifting, all of which significantly mediated self-reported social anxiety symptoms. Although we did not specifically measure these variables, our study suggests that significant symptom change in MBCT may also be due to enhancement of positive, approach related affect, which is theoretically and conceptually distinct from the findings of Goldin and Gross (2010), which highlight mechanisms of action that are similar in principle to those observed in CBT and GCBT (Goldin et al., 2016).

The results of the current pilot study should be evaluated in light of study limitations. First, given the pilot nature of the study, our sample size was small. When considering the impact of small sample sizes on the stability and accuracy on estimated regression coefficients in multivariate linear models such as ours, we note that prior Monte Carlo simulations (Austin and Steyerberg, 2015) have suggested a minimum number of subjects per variable of at least two in order to produce unbiased estimates of coefficients and confidence intervals. Our hierarchical linear models included five predictors, which therefore suggests a minimum total N of at least 10 cases. Although our GAD and SAD-only groups both exceeded the minimum recommended cutoff for unbiased estimation of regression coefficients (*N* = 15, and 14, respectively), we nevertheless note that future studies should consider a larger sample size to minimize the risk of Type 2 errors. In addition, for the regression analyses in the reduced samples, a substantial number of individuals met criteria for both SAD and GAD and thus were included in both reduced sample analyses. Prior work has illustrated that rates of comorbidity between primary SAD and GAD range between 23.8% (Mennin et al., 2000) and 36% (Brown et al., 2001). Thus, continued examination of this frequent pattern of comorbidity remains an important area for future work, particularly as it relates to the mechanisms by which PA deficits may influence its etiology and course. Although the preliminary nature of this study limits definitive conclusions regarding short- and long-term changes in GAD symptoms for patients reporting primary and secondary comorbid diagnoses, results of this small trial do highlight the possibility that SAD and GAD symptom reduction may be associated with distinguishable changes in positive versus NA (respectively) in a mindfulness-based intervention such as MBCT.

An additional concern when conducting a relatively large number of statistical tests pertains to the increased risk for type I error. To address this issue we increased the stringency of the critical p value according to the number of experiment-wide statistical tests, adjusted for false discovery rate (FDR). Using the approach presented in Curran-Everett (2000), the 48 tests reported in this paper would suggest an adjusted critical *p*-value of 0.011. If taking this approach, the majority of statistical tests in support of the hypotheses would no longer meet significance, see **Tables 5–8** for list of tests reported with corresponding adjusted significance levels. Because of this, we recommend that our results should be interpreted with caution in recognition of the exploratory nature of the study.

**Table 5:** Hierarchical regressions in full sample, with adjusted critical *p*-value.

Predicted variable	Predictors in Set	Δ*R*^2^	Δ*F*	β	*t*	*p*
**Post-LSAS-SR**						
Block 1		0.58	8.13			0.001
	Pre-LSAS			0.77	4.13	0.001
	Pre-PANAS:PA			-0.07	-0.42	0.677
	Pre-PANAS:NA			0.14	0.49	0.631
Block 2		0.04	1.55			0.230
	Δ PANAS:NA			-0.14	-0.60	0.558
§Block 3		0.10	5.30			0.035
	Δ PANAS:PA			0.34	2.30	0.035
**Post-PSWQ**						
Block 1		0.55	7.31			0.002
	Pre-PSWQ			0.27	1.22	0.241
	Pre-PANAS:PA			-0.01	-0.05	0.961
	Pre-PANAS:NA			0.76	2.45	0.026
§Block 2		0.15	8.05			0.011
	Δ PANAS:NA			-0.62	-2.65	0.018
§Block 3		0.08	6.04			0.026
	Δ PANAS:PA			0.30	2.46	0.026

*§Denotes statistical tests that are no longer significant after the application of the False Discovery Rate (FDR) adjusted *p*-value (0.011). For each regression block, the *p*-value is the significance of change in predicted variance. For each predictor, the p value is the significance of that individual predictor. The β, *t*, *p* values for each predictor are the values in the final step of the regression. Pre, Before treatment; Post, After treatment; LSAS-SR (Liebowitz Social Anxiety Scale – Self-Report; Liebowitz, 1987); PSWQ (Penn State Worry Questionnaire – Self-Report; Meyer et al., 1990); PANAS, PA/NA (Positive Affect/Negative Affect subscales of Positive Affect and Negative Affect Schedule; Watson et al., 1988).*

**Table 6:** Hierarchical regressions in reduced samples (SAD and GAD), with adjusted critical *p*-value.

Predicted variable	Predictors in Set	Δ*R*^2^	Δ*F*	β	*t*	*p*
**Reduced Sample: SAD**					
Post-LSAS						
Block 1		0.33	1.61			0.249
	Pre-LSAS			0.46	1.87	0.098
	Pre-PANAS:PA			-0.09	-0.39	0.705
	Pre-PANAS:NA			0.25	0.56	0.588
Block 2		0.11	1.66			0.230
	Δ PANAS:NA			-0.31	-0.80	0.444
§Block 3		0.30	8.72			0.018
	Δ PANAS:PA			0.58	2.95	0.018
**Reduced Sample: GAD**					
Post-PSWQ					
Block 1		0.66	7.09			0.006
	Pre-PSWQ			0.012	0.39	0.705
	Pre-PANAS:PA			0.26	1.36	0.208
	Pre-PANAS:NA			0.86	1.91	0.088
§Block 2		0.15	7.55			0.021
	Δ PANAS:NA			-0.84	-2.43	0.038
Block 3		<0.01	0.14			0.714
	Δ PANAS:PA			0.06	0.38	0.714

*§Denotes statistical tests that are no longer significant after the application of the False Discovery Rate (FDR) adjusted p-value (0.011). For each regression block, the p value is the significance of change in predicted variance. For each predictor, the p value is the significance of that individual predictor. The β, t, p values for each predictor are the values in the final step of the regression. SAD, Individuals with social anxiety disorder diagnosis; GAD, Individuals with generalized anxiety disorder diagnosis; Pre, Before treatment; Post, After treatment; LSAS-SR (Liebowitz Social Anxiety Scale – Self-Report; Liebowitz, 1987); PSWQ (Penn State Worry Questionnaire – Self-Report; Meyer et al., 1990); PANAS, PA/NA (Positive Affect/Negative Affect subscales of Positive Affect and Negative Affect Schedule; Watson et al., 1988).*

**Table 7:** Linear regressions predicting positive affect change from mindfulness measures.

Predictors	*R*^2^	*F*	β	*t*	*p*
**Linear Regressions**				
§Δ FFMQ: Awareness	0.26	6.84	0.51	2.62	0.017
Δ FFMQ: Total	0.18	4.32	0.42	2.08	0.051
**Multiple Linear Regression**				
Overall model	0.36	1.83			0.163
Δ FFMQ: Awareness			0.39	1.28	0.22
Δ FFMQ: Observing			<-0.01	-0.02	0.99
Δ FFMQ: Describing			-0.23	-1.05	0.31
Δ FFMQ: Non-judging			0.13	0.42	0.68
Δ FFMQ: Non-reacting			0.24	0.57	0.58

*§Denotes statistical tests that are no longer significant after the application of the False Discovery Rate (FDR) adjusted *p*-value (0.011). PANAS, PA (Positive Affect Scale of Positive Affect and Negative Affect Schedule; Watson et al., 1988). FFMQ (Five Facet Mindfulness Questionnaire – Self-Report; Baer et al., 2008). Total corresponds to the total FFMQ total scale. The predictors in the multiple linear regression are the subscales of the FFMQ.*

**Table 8:** Changes in self-report variables, with adjusted critical *p*-value.

		Pre-MBCT	Post-MBCT	*t*-value	*p*-value	Effect size
Measure/Sample	*N*	*M* (*SD*)	*M* (*SD*)	*t*	*p*	*d*
**LSAS-SR**						
Full-Sample	22	60.95 (29.25)	47.68 (23.63)	3.26	0.004	0.45
§SAD	14	68.86 (25.54)	52.86 (22.22)	2.65	0.020	0.63
**PSWQ**						
Full-Sample	22	52.05 (16.06)	45.36 (14.54)	2.81	0.010	0.42
GAD	15	54.33 (14.34)	46.40 (15.44)	3.02	0.009	0.55
**PANAS: PA**						
Full-Sample	22	20.41 (8.59)	24.59 (10.46)	-3.06	0.006	0.49
§SAD	14	20.64 (9.54)	25.50 (11.89)	-2.85	0.014	0.51
§GAD	15	19.47 (7.97)	23.07 (7.78)	-2.57	0.022	0.45
**PANAS: NA**						
§Full-Sample	22	13.27 (10.57)	10.23 (7.80)	2.12	0.046	0.29
SAD	14	15.57 (11.96)	11.29 (7.61)	2.08	0.058	0.36
GAD	15	14.47 (11.61)	10.60 (8.25)	2.05	0.060	0.33
**FFMQ: Awareness**						
Full-Sample	22	24.86 (6.79)	28.00 (6.36)	-2.90	0.009	0.46
§SAD	14	24.43 (6.66)	28.43 (6.42)	-2.67	0.019	0.60
§GAD	15	24.00 (7.45)	26.73 (6.87)	-2.05	0.059	0.37
**FFMQ: Total**						
Full-Sample	22	109.86 (17.46)	123.50 (21.50)	-3.64	0.002	0.78
SAD	14	109.07 (17.08)	129.93 (18.82)	-4.95	<0.001	1.22
§GAD	15	108.67 (17.88)	120.87 (18.31)	-2.51	0.025	0.68

*§Denotes statistical tests that are no longer significant after the application of the False Discovery Rate (FDR) adjusted *p*-value (0.011). SAD, Individuals with social anxiety disorder diagnosis; GAD, Individuals with generalized anxiety disorder diagnosis; LSAS-SR (Liebowitz Social Anxiety Scale – Self-Report; Liebowitz, 1987); PSWQ (Penn State Worry Questionnaire – Self-Report; Meyer et al., 1990); PANAS, PA/NA (Positive Affect/Negative Affect subscales of Positive Affect and Negative Affect Schedule; Watson et al., 1988); FFMQ, Awareness/Total (Awareness subscale and Total scale of the Five Facet Mindfulness Questionnaire – Self-Report; Baer et al., 2008); Effect size (d) = difference in means/pre-treatment standard deviation.*

Another limitation of our study was the lack of a comparison condition, which prohibits conclusions regarding the comparative efficacy of MBCT in relation to more established treatments such as CBT (such as the results reported in Piet et al., 2010), and also prevents any conclusions regarding differential mechanisms of action. For example, the presence of a CBT comparison condition could have illustrated differential mechanisms of action in these interventions, with the expectation that CBT and exposure-based interventions more generally might operate on well-delineated fear and arousal systems by promoting fear extinction and habituation, whereas MBCT might be expected to act exclusively on positive, approach related motivational deficits. An additional limitation is that our study relied on self-report, and did not include other measures such as ecological assessment or measures of general wellbeing. Our study also did not include a measure of treatment adherence (such as extent of homework compliance) or follow-up outcomes, which therefore limits our ability to examine whether symptom change is a direct result of treatment involvement and whether symptom change is maintained after treatment. Despite these limitations, the current study provides the first evidence for MBCT as a new model approach for the treatment of social anxiety symptoms through mechanisms that are likely to be distinct from traditional exposure-based interventions, which instead focus on fear extinction and habituation processes as their central mechanisms of action.

## Author Contributions

MS coordinated the data collection and led the manuscript development process. DS assisted in data collection and study coordination. LB assisted in analysis of data. AV contributed to the manuscript development process. JR oversaw the development and implementation of the study, and contributed to the manuscript.

## Conflict of Interest Statement

The authors declare that the research was conducted in the absence of any commercial or financial relationships that could be construed as a potential conflict of interest.
